# Rare Giant Infected Intradiploic Skull Epidermoid Cysts

**DOI:** 10.7759/cureus.29375

**Published:** 2022-09-20

**Authors:** Joshua S Catapano, Rohin Singh, Michael T Lawton, Shawn M Stevens, Randall W Porter

**Affiliations:** 1 Department of Neurosurgery, Barrow Neurological Institute, Phoenix, USA

**Keywords:** infection, tumor, giant intradiploic epidermoid, epidermoid, cyst

## Abstract

Infections associated with giant intradiploic cranial epidermoid cysts are rare. This case report describes the successful surgical management of a 71-year-old diabetic man with a giant intradiploic cranial epidermoid cyst associated with a secondary infection. The patient underwent successful resection of the infected lesion with washout, debridement, and obliteration of the eustachian canal and external auditory canal. At the six-month follow-up, the infection was resolved and the patient was doing well clinically. Intradiploic epidermoid cysts are rare, and the presence of a superimposed otogenic infection is exceptionally rare and infrequently reported in the neurosurgical literature.

## Introduction

Cranial epidermoid cysts are encountered infrequently, accounting for 0.2% to 1% of intracranial tumors [1]. Intradiploic epidermoid tumors represent 25% of these lesions [2]. Congenital epidermoid cysts arise from the improper implantation of ectoderm during neural groove or epithelial fusion line closure, whereas acquired cysts arise secondary to trauma to surface epithelium [1]. Two cases of iatrogenically induced intradiploic epidermoid cysts have been reported, one following a strip craniectomy and another following an intradermal melanocytic nevus [3]. Although these cysts are typically benign, cases of malignancy have been reported; headaches and seizures are the most common presenting symptoms [1,4-6]. A literature review found 31 reports of giant intradiploic epidermoid cysts, of which only three were infected [1-20].

## Case presentation

**Figure 1:**
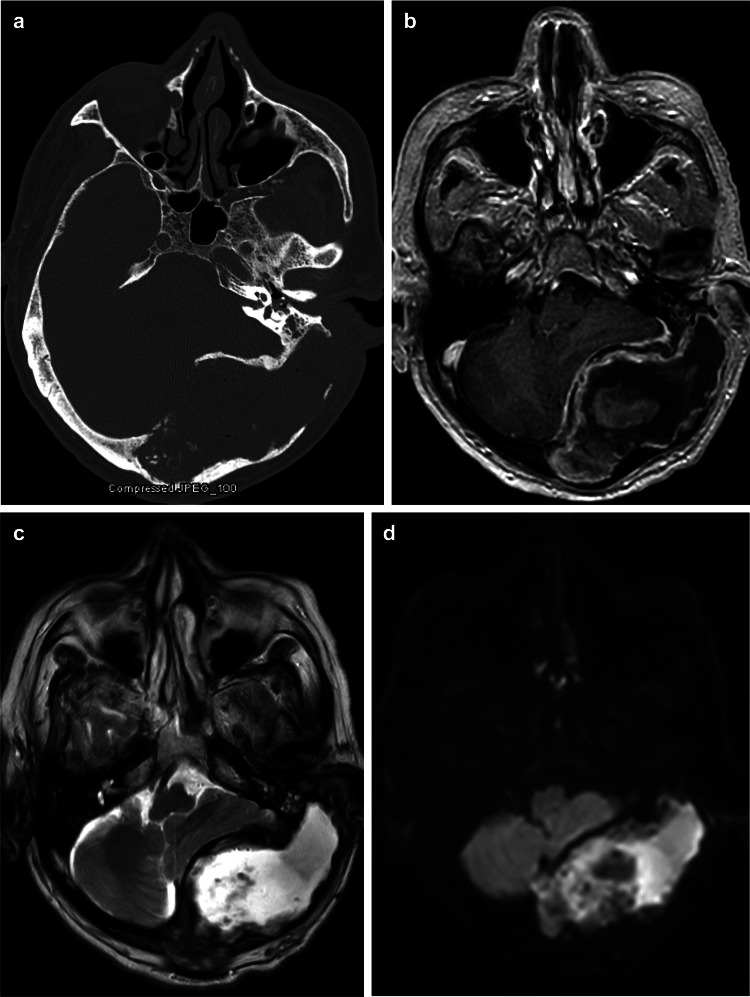
Preoperative imaging. (a) Axial computed tomography showing erosion of the external auditory canal and posterior fossa with an intradiploic lesion in the occiput. Axial magnetic resonance imaging (T1-weighted image) with gadolinium showing rim enhancement (b), T2-weighted image showing an isointensity to cerebral spinal fluid in the lesion (c), and diffusion-weighted image showing bright spots suspicious for an epidermoid tumor with possible infection (d). *Used with permission from Barrow Neurological Institute, Phoenix, Arizona*.

A 71-year-old diabetic man presented with dizziness, low-grade fever, and chronic purulent otorrhea. He had a secondarily infected intradiploic mass arising from the occipital bone with erosion into the mastoid space and gross bony dehiscence of both the external auditory canal (EAC) and posterior fossa plate on computed tomography. Imaging showed occlusion of the left jugular vein and sigmoid and transverse sinuses, rim enhancement, diffusion-weighted imaging restriction, and significant mass effect upon the left cerebellum (Figure 1).

The patient underwent surgery using a left transtemporal-transoccipital approach for washout and near-total resection of the mass (Video 1).

Video 1: A left transtemporal-transoccipital approach for washout and near-total resection of an infected giant intradiploic epidermoid tumor.

The external auditory canal was over-closed, and all remaining epithelial elements, including the tympanic membrane and middle ear mucosa, were removed. The eustachian tube was obliterated, and the facial nerve was preserved throughout its course. *Used with permission from Barrow Neurological Institute, Phoenix, Arizona.*

Gross inspection revealed packed epithelial debris punctuated with microabscesses throughout. Infection transmission had occurred via erosion of the lesion through the bony EAC. Ultimately, after debulking, all visible squamous epithelial elements were stripped. Histopathology was consistent with epidermoid tumor and secondary inflammatory processes. To prevent a recurrence, the EAC was overclosed, and all remaining epithelial elements, including the tympanic membrane and middle ear mucosa, were removed. The eustachian tube was obliterated. The facial nerve was preserved throughout its course. Care was taken to keep the dissection extradural in regions of gross bony dehiscence, and a cerebrospinal fluid leak was avoided. After copious debridement and irrigation with bacitracin solution, the defect was coated with vancomycin powder. The cavity was obliterated in part with muscle turn-in flaps.

**Figure 2:**
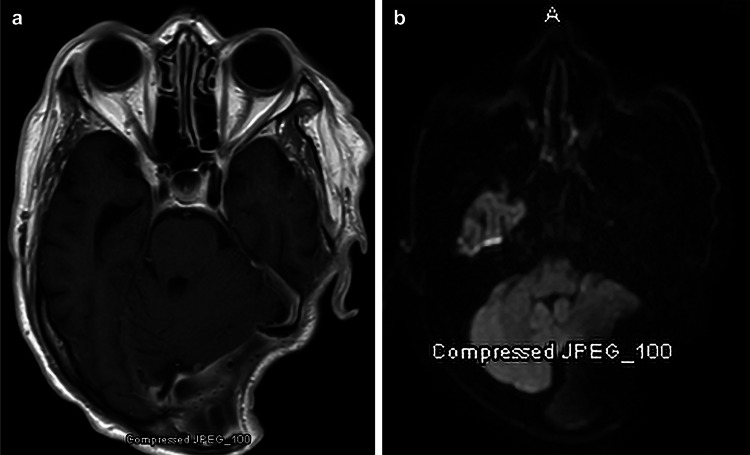
Six-month postoperative axial magnetic resonance imaging. (a) No signs of infection and near-complete tumor removal shown on T1-weighted image with gadolinium and (b) diffusion-weighted image. *Used with permission from Barrow Neurological Institute, Phoenix, Arizona.*

Postoperative magnetic resonance imaging demonstrated heterogeneous tissue enhancing over the resection site and no infectious signs. At six months, the patient had near-complete tumor resection with no signs of infection (Figure 2); he had left-sided sensorineural hearing loss, as expected, but no other serious deficits or complications from surgery.

The patient gave informed consent for the case report, and the institutional review board at St. Joseph’s Hospital and Medical Center (Phoenix, AZ) approved the study protocol.

## Discussion

Intradiploic epidermoid cysts are extremely rare and slow-growing brain tumors. These cysts commonly arise in the third or fourth decade of life as painless, well-circumscribed, subcutaneous lesions and are more common in men. Epidermoid cysts can result from abnormal embryonic development, or they can be secondary to trauma that traps epidermal tissue in the diploe of the skull. The location and cause of the tumor may lead to variable surgical complications and affect the optimal treatment modality for lesions, as in the case described above. The diagnosis of these lesions is usually suggested via imaging and confirmed through histological examination.

Jaiswal and Mahapatra [5] reported on eight patients with giant intradiploic epidermoid cysts. Three patients had superimposed infections, similar to our patient, whereas five presented with only local swelling and pain. Seven patients had complete surgical resections, and one had a near-complete resection. The three patients with infection required additional antibiotics and infection-control measures before surgery. All patients received corticosteroids before and after the operation and a 7- to 10-day course of postoperative antibiotics. One patient was treated again for a surgical wound infection, and all patients were followed up for one to three months with no reported recurrences.

Extra attention should be given to preventing the infection of giant epidermoid cysts because of the likelihood of exposure to the external environment. Giant epidermoid cysts frequently occur within the frontal and temporal bones and can easily be exposed to pathogens through the external auditory meatus, orbit, or cribriform plate [19]. Duan et al. [9] recommended repeated washing of the tumor cavity with 0.9% saline to prevent surgical-site infections and meningitis. Primary or secondary infection can lead to extensive damage and long-term deficits, which should be a key consideration in giant epidermoid cyst removal.

Because of their size and location, giant intradiploic epidermoid cysts often have extensive attachments to the surrounding bone and dura. Therefore, careful curettage is often necessary to fully liberate the tumor capsule from its dural attachments [4,5]. Most patients with giant epidermoid cysts undergo complete resection, and despite the invasive nature of these tumors, removal often results in little to no long-term deficit [9,11,14,15,17-19].

Recurrence of epidermoid and giant epidermoid cysts has been reported, usually in patients with incomplete resection [14]. In addition, malignant transformation of recurrent tumors is common and usually develops as squamous cell carcinoma [14]. These transformations are associated with a poor prognosis, even with resection, radiotherapy, and chemotherapy [1,10,12,14]. Consequently, initial treatment should be complete resection to avoid adverse outcomes.

Complete resection remains the treatment of choice for epidermoid cysts. Although patients can live for many years with these tumors, often asymptomatically, early surgery is most effective in preventing complications associated with tumor growth. This is a relatively safe procedure, even within a young patient population [11].

## Conclusions

Intradiploic epidermoid cysts are rare, and the presence of a superimposed otogenic infection is exceptionally rare and infrequently reported in the neurosurgical literature. We present a case involving epidermoid resection with infection down to the sternocleidomastoid and EAC. Each intradiploic epidermoid case is unique, and careful examination of the tumor’s relationship with the patient’s anatomy is essential before treating difficult lesions. Some complications may be unpredictable, and the surgeon must be meticulous in the analysis and approach for proper care.
